# High Efficiency and Low Migration Hyperbranched Silicone Contain Macrophotoinitiators for UV-Cured Transparent Coatings

**DOI:** 10.3390/polym12123005

**Published:** 2020-12-16

**Authors:** Yunxin Fan, Yan Song, Na He, Fei Cheng, Xiaojiao Jiao, Guoqiao Lai, Xilin Hua, Xiongfa Yang

**Affiliations:** Key Laboratory of Organosilicon Chemistry and Material Technology of Education Ministry, College of Material, Chemistry, and Chemical Engineering, Hangzhou Normal University, Hangzhou 311121, China; 15933726811@163.com (Y.F.); sssyhznu@163.com (Y.S.); hena960827@163.com (N.H.); cf123333@163.com (F.C.); jiaojiao96126@163.com (X.J.); laiguoqiao@aliyun.com (G.L.); louxh@aliyun.com (X.H.)

**Keywords:** UV-cured coating, hyperbranched silicone containing macrophotoinitiators, migration, the tensile strength

## Abstract

A kind of hyperbranched silicone containing macrophotoinitiators (HBSMIs) were synthesized from 2-hydroxy-2-methyl-1-phenyl propanone (HMPP) and the UV-curing behaviors of HBSMIs were investigated in UV-cured transparent polyurethane-acrylate (PUA) coatings. HBSMIs show higher UV-initiating efficiency than HMPP. The migration of HBSMIs from the UV-cured coatings can be as low as 1.7–6.0 wt%, which is obviously lower than the migration of HMPP. There is a remarkable improvement of the tensile strength of the UV-cured materials initiated by HBSMI in comparison to that of the materials prepared with the same PUA initiated by HMPP. Especially for the UV-cured materials prepared from PUA with 20 wt% 1,1,1-tris(hydroxymethyl)propane (TMP), the tensile strength and the strain at break increased from 6.81 MPa to 12.14 MPa and from 43.0% to 71.9%, respectively. The fraction of improvement for the tensile strength and the strain at break is as high as 78.9% and 67.2%, respectively. The coatings prepared with HBSMI also have better UV resistance ability than those coatings prepared with HMPP because they turn slightly yellow when they are aged by UV for about 15 min while the coating prepared with 4 wt% of HMPP will turn yellow only aged by UV for 2 min. These results suggest that preparation hyperbranched silicone containing macrophotoinitiators will be one of the good strategies to improve the curing efficiency of the UV-curing system, reduce the migration of UV initiator from cured material, improve the mechanical and UV resistance performance of UV-cured materials.

## 1. Introduction

UV-curing technology has drawn more and more attention in many fields such as coatings [1,2,3,4,5], 3D printing [6,7], biomaterials [8] because of its environmentally friendly and high efficiency advantages. A conventional UV-curing system usually is a composition of UV-curable polymers, reactive monomers, additives, and a photoinitiator or photoinitiator system [9]. The UV initiator or initiator system is critical because it determines the UV-curing speed and significantly affects the comprehensive performance of cured materials such as yellowing, odor, migration, etc., [9,10,11,12]. Small molecule UV photoinitiators such as benzoin, acetophenone, α-hydroxy ketone, benzophenone, thioxanthone, and their derivatives have been widely used [6,8,9,10,11,12], however, their obviously disadvantages such as relatively strong odor and high migration from cured films greatly limit their applications [10,13,14,15,16,17,18]. Especially, the unreacted small molecule photoinitiator molecules and photolysis products can migrate to the surface of post-cured material or may be extracted into liquid-packed goods when the UV-cured materials were applied in food packaging [14,19].

Conversely, macromolecular UV initiators of the corresponding low molecular weight analogs have been proved to show obvious advantages of lower odor, migration, toxic while better yellowing resistance [10,13,14,15,16,17,18]. Furthermore, the UV-initiated efficiency of them can be enhanced for the better solubility and compatibility than the low molecular weight analogs [20]. Therefore, numerous strategies have been developed to design novel macromolecular UV initiators [15,16,17,18,19]. 

As a kind of low-cost and high-efficient photoinitiator, 2-hydroxy-2-methyl-1-phenyl propanone (HMPP) has been widely utilized to design macrophotoinitiators by the reactions of the hydroxy groups [21,22,23,24,25,26]. For example, Xie prepared amphiphilic macrophotoinitiators with HMPP and it reveals that the photolysis rate of them was slightly lower than that of HMPP, but the migration rate of them from the UV-cured material was lower than that of HMPP [21]. In the past decades, many efforts have been paid to develop novel silicone macrophotoinitiators with HMPP because they not only possess low migration and odor, but also can effectively reduce the oxygen inhibition and improve properties of UV-cured materials [27,28,29,30,31]. A novel hydrosoluble photocleavage polysiloxane photoinitiator based on HMPP and aminopolysiloxane for preparing a gradient polymer shows relatively good solubility in water and excellent photoinitiating efficiency [23] and a photocleavage polymerizable organosilicon macromolecular photoinitiator based on HMPP exhibits the migration of photolysis fragments toward the cured material surface was mitigated significantly [24]. A kind of water-borne polysiloxane-modified photoinitiator based on HMPP can mitigate the oxygen inhibition and the cured materials have better mechanical properties and thermostability compared with that by HMPP [25]. These good works promote the research and application of HMPP-based silicone macrophotoinitiators. However, these silicone macromolecular photoinitiators usually have poor compatibility with the UV-curable polymers, which will result in a lower transparency and relative poor mechanical of UV-cured products [4]. As we know, high optical transmittance materials are extensively sought, especially for optics and “invisible” wearable sensors [32,33], which promotes us to explore new silicone containing macromolecular UV initiators for UV-cured transparent materials. 

Hyperbranched polymers have relatively lower viscosity and fairly good compatibility with the other polymers compared to their linear analogues [34,35]. Therefore, design hyperbranched silicone macrophotoinitiators might be a good choice to improve the compatibility of silicone macrophotoinitiators with the UV-curable polymers. Recently, a kind of hyperbranched poly(siloxysilane) macrophotoinitiators were prepared with HMPP and the results showed that the macrophotoinitiators have higher efficiency than HMPP and the thermal stability of UV-cured materials prepared with macrophotoinitiators is higher than that of materials cured with HMPP [26]. In this paper, a kind of hyperbranched silicone containing macrophotoinitiators based on HMPP were synthesized and the UV-curing behaviors were investigated in UV-cured transparent polyurethane-acrylate coatings. Compared with HMPP, the hyperbranched silicone containing macrophotoinitiators show fairly high UV-initiating efficiency and low migration. The mechanical performance and UV-resistant ability of the coatings prepared with the macrophotoinitiators are superior to that of the coatings prepared with HMPP. 

## 2. Materials and Methods

### 2.1. Materials

2-Hydroxy-2-methyl-1-phenyl propanone (HMPP, 99.0%, AR) was purchased from Chitec Technology Co., Ltd., (Shanghai, China). 3-Isocyanatopropyltriethoxysilane (95.0%, AR) was bought from Aladdin Chem. Co., Ltd., Shanghai, China. Polytetramethylene ether glycol (PTMG, Mn = 2000, 96.0%, AR), neopentyl glycol (NPG, AR), isophorone diisocyanate (IPDI, 99.0%, AR), ditin butyl dilaurate (DBTDL, 95.0%, AR), 1,1,1-tris(hydroxymethyl)propane (TMP, 98.0%, AR), and hydroxypropyl acrylate (97.0%, AR) were purchased from Beijing HWRK Chem. Co., Ltd., Beijing, China. Various polyurethane-acrylates (PUAs) were synthesized according to the work reported by our group (Table 1) [2].

### 2.2. Synthesis of Hyperbranched Silicone Containing Macrophotoinitiators (HBSMIs)

**Scheme 1:**
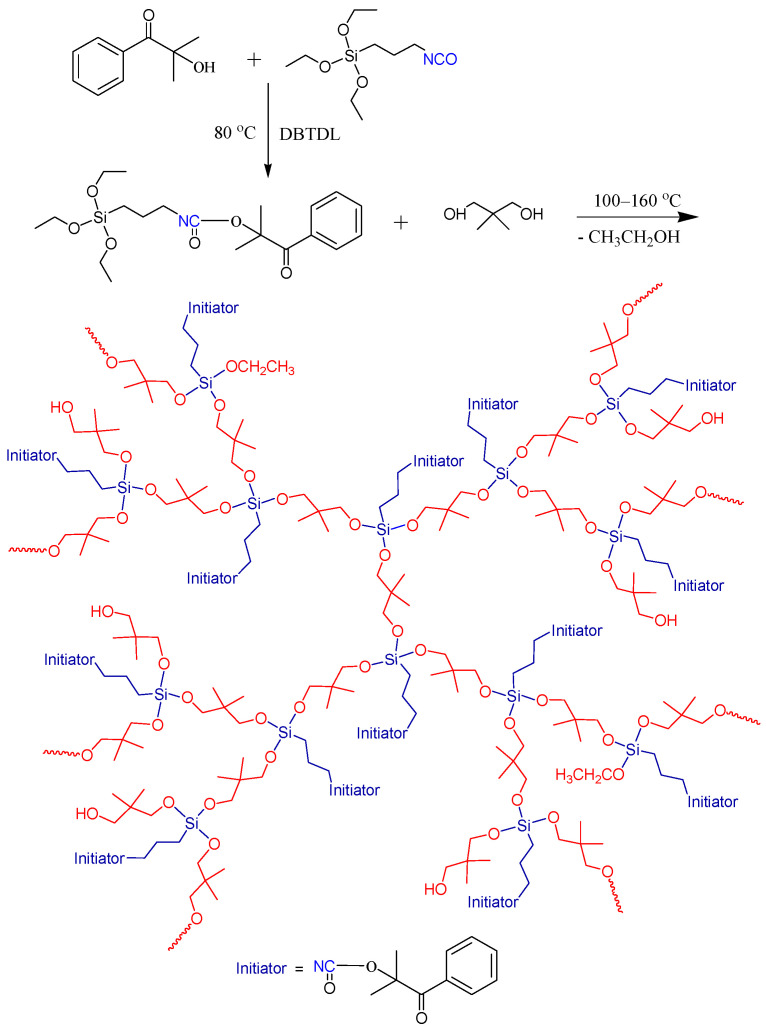
The synthesis route for hyperbranched silicone containing macrophotoinitiators (HBSMI).

HBSMIs prepared with HMPP were synthesized according to Scheme 1 [36,37]. A typical procedure for the preparation of HBSMI is as following: 24.70 g 3-isocyanatopropyltriethoxysilane (0.1 mol), 16.42 g HMPP (0.1 mol), and 0.12 g DBTDL were added into a 100 mL three-necked flask equipped with a thermometer, a top stirrer, a N_2_ gas inlet and kept at 80 °C for 6 h and a triethoxysilane with HMPP substitute was obtained. Subsequently, NPG (As shown in Table 2) was added and the mixture was heated and kept around 100 °C until some distillate was distilled off. Then the mixture was heated to about 160 °C and kept the distillate temperature below 78 °C until the distillate temperature dropped below 55 °C. Subsequently, the residual small molecules were taken off under 110 °C/130 mmHg and a transparent liquid of HBSMI was prepared. The ^1^H–NMR, ^29^Si–NMR, and SEC curve of products are shown in Figures S1, S2, and S3, respectively. 

^1^H–NMR spectrum (400 Hz, shown in Figure S1): 3.96–3.81, 3.80–3.60, and 3.56–3.40 ppm are assigned to the protons of –SiOC*H*_2_C(CH_3_)_2_C*H*_2_OSi– and HOCH_2_C(CH_3_)_2_C*H*_2_OSi–, HOC*H*_2_C(CH_3_)_2_CH_2_O–, –SiOC*H*_2_CH_3_, respectively [1,36,37]. 7.52–7.15 ppm is assigned to the proton of –C_6_*H*_5_ in HMPP substitute. 1.0–0.70 ppm is assigned to the protons of –C*H*_3_ in HMPP substitute, –SiOCH_2_C*H*_3_, –SiOCH_2_C(C*H*_3_)_2_CH_2_OSi–, and HOCH_2_C(C*H*_3_)_2_CH_2_OSi–. 1.57–1.40, 2.43–2.30, and 1.19–1.0 ppm are assigned to the protons of –SiCH_2_C*H*_2_CH_2_NCO–, –SiCH_2_CH_2_C*H*_2_NCO–, and –SiC*H*_2_CH_2_CH_2_NCO–, respectively.

^29^Si-NMR spectrum of the HBSMI is obtained, as shown in Figure S2. The chemical shifts at −59.3 and −68.1 ppm can be attributed to the complete branched and the incomplete branched Si respectively [36,37]. The broad peak in the range of −75.0 ppm–−125.0 ppm is assigned to the Si in quartz NMR tube.

Figure S3 reveals the SEC analysis for HBSMI–5 prepared. It can be seen that the average number average molecular weight (*M*_n_) of HBSMI–5 is about 6.88 × 10^3^ Da, molecular weight distribution is PDI = 2.90, and the α constant of Mark–Houwink–Sakurada is 0.178(±3.64%), which means the HBSMI obtained is a kind of hyperbranched polymer.

### 2.3. Preparation of Ultraviolet (UV)-Cured Coatings

**Scheme 2:**
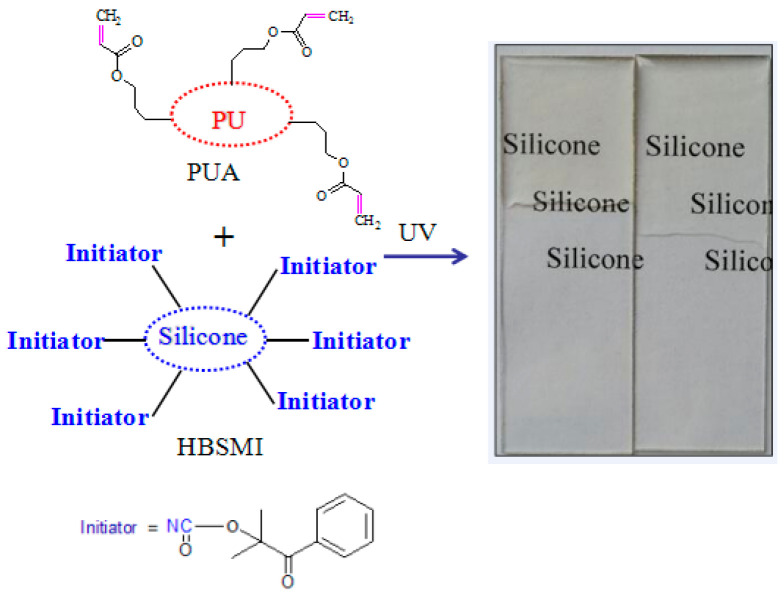
Preparation of UV-cured materials.

The UV-cured coatings were prepared according to the procedure shown in Scheme 2. About 0.4 g homogeneous mixtures of PUAs and HBSMI were heated to about 60 °C in an oven and then deposited on about half of the glass slides and kept away from light for 24 h under ambient temperature to let the mixtures smooth as possible, finally transparent coatings with thickness about 0.5 mm were prepared after being cured by UV (ZB1000, Changzhou Zibo Electron Technology Co., Ltd., Jiangsu, China). Laser wavelength 365 nm, Radiation intensity 10.6 mw.cm^−2^, the distance of the slides to the light is 20 cm). If about 1.6 g homogeneous mixtures PUAs and HBSMI were heated to about 60 °C in an oven and then deposited on total of the glass slides and kept away from light for 24 h at ambient temperature, the UV-cured coatings with thickness about 0.7 mm were prepared. These coatings can be peeled off from the glass slides to prepare film samples for mechanical experiments.

### 2.4. Characterization

All the NMR analyses were conducted in CDCl_3_ without tetramethylsilane (TMS) as internal reference in quartz NMR tube. ^1^H-NMR spectrum was recorded on a Bruker AVANCE AV400 (400 MHz, Bruker, Karlsruhe, German) spectrometer, while ^29^Si-NMR spectrum was recorded on a Bruker AVANCE AV600 (600 MHz, Bruker, Karlsruhe, German) spectrometer. Fourier-transform infrared (FT-IR) spectroscopic analysis of coatings scraped from glass slides was operated on a Nicolet 700 spectrometer (Nicolet, Madison, WI, USA). Thermal properties of the coatings were examined by thermogravimetric analysis (TGA, TG 209C, Netzsch, Free State of Bayern, German), in which samples were heated from ambient temperature to 800 °C at a rate of 10 °C min^−1^ in a N_2_ atmosphere. SEC curve of HBPSH was recorded on a Waters 1515 (Waters, Worcester County, MA, USA) with THF as fluent using polystyrene as standard. The tensile test of the films (6 mm × 0.7 mm × 8 mm strips) scraped from the slides was carried out according to GB/T 528–2009/ISO 37:2005 on an UH6503D electronic tensile testing machine (Optimal Hung Measurement & Control Technology Co. Ltd., Shanghai, China). The load is 100 N with a loading rate of 80 mm/min. The powders of coatings obtained were placed in acetone at room temperature for 48 h and then kept at 60 °C/20 mmHg for 24 h, the degree of curing contents were calculated as a percentage of the residual mass in the original mass of the powders. The migration rate of UV initiator in coatings was investigated by placing the coatings in water at room temperature for 24 h and then kept at 60 °C/20 mmHg for 24 h, then the degree of migration was calculated as a percentage of the loss mass in the original mass of the coatings. Transmittance, pencil hardness, adhesion property were measured according to references [1,2,3,4,5].

## 3. Results and Discussion

### 3.1. Effect of UV-Curing Time on the UV-Cured Coatings Initiated by HBSMI and HMPP

**Figure 1:**
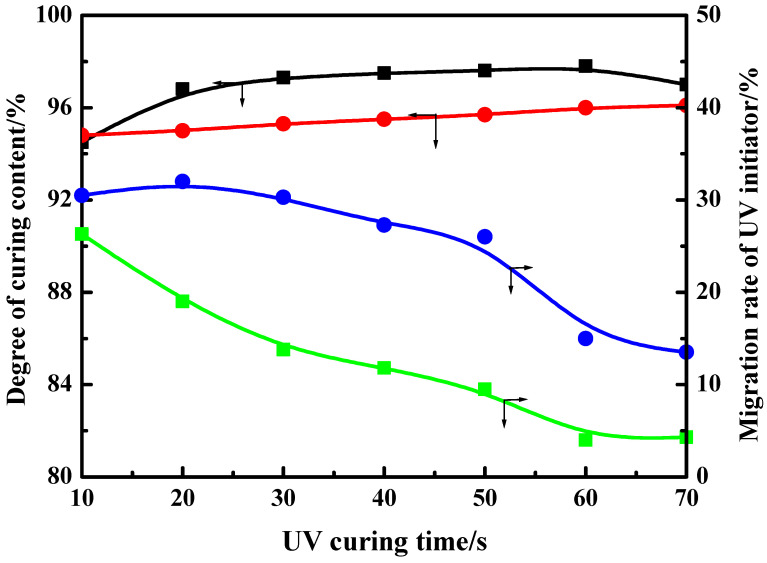
The comparation of degree of curing content of coatings and the migration rate of UV initiator from coatings cured by HBSMI (■) and HMPP (●). Conditions: PUA is PUA–3 and the amount of UV initiator is 4 wt% of PUA–3.

To compare the UV-cure behavior of HBSMI and HMPP, the effect of UV-curing time on the UV-cured coatings initiated by HBSMI and HMPP was studied and shown in Table 1 and Figure 1. When the amount of HBSMI and HMPP is 4 wt% of PUA respectively, it can be obviously seen from Table 3 and Figure 1 that the degree of curing content and pencil hardness of the coatings cured by HBSMI for the same time are higher than those coatings cured by HMPP respectively. It might suggest that the UV-initiated efficiency of HBSMI is much higher than HMPP because of the solubility and compatibility of HBSMI superior to HMPP, which is similar to the work reported previously [20]. The fairly high UV-initiate efficiency of HBSMI is also can be proved by FT–IR spectra of coatings cured for various time initiated by HBSMI shown in Figure S4 because even the coatings were cured only for 10 s, the characteristic absorption peaks of acrylate in PUA are vanished.

It is exhibited in Figure 1 that the migration rate of HBSMI is obviously lower than that of HMPP when the coatings were cured at the same conditions. The migration rate of HBSMI is not higher than 6.0 wt% while that of HMPP is about 14 % when the UV systems were cured for 60 s. In comparison to the migration rate of the other macrophotoinitiators reported, the mass fraction of silicone-thioxanthon reported by Tang is as low as 8.1 % [30], and the mass fraction of a series of novel UV-curable macrophotoinitiator based on Irgacure 184 reported by Qu is in the range of 10.9–19.6 wt% [38]. Therefore, a conclusion can be drawn that the migration rate of HBSMI is fairly low.

### 3.2. Mechanical Performance of the UV-Cured Coatings Prepared with Various of PUAs Initiated by HBSMI and HMPP

**Figure 2:**
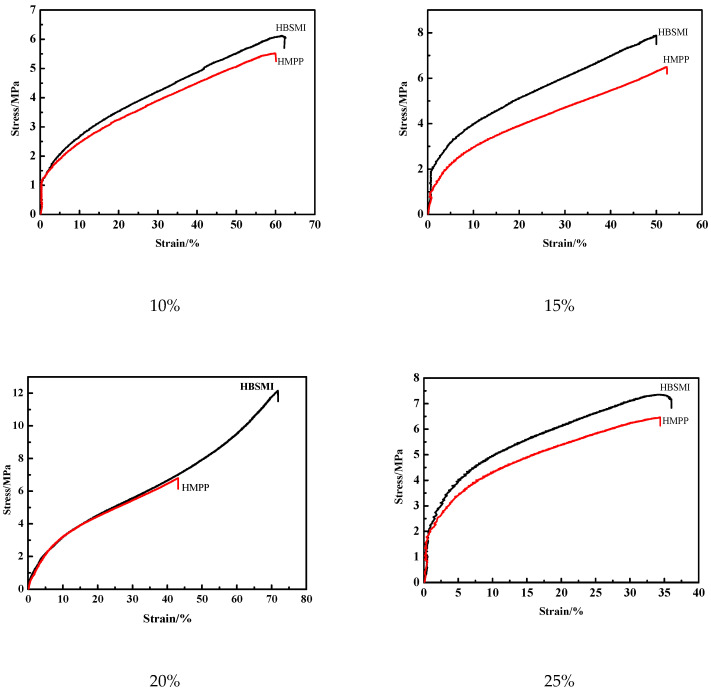
The effect of various amount of 1,1,1-tris(hydroxymethyl)propane (TMP) on the mechanical performance.

The comparison study of mechanical performance of the UV-cured coatings prepared with several PUAs initiated by HBSMI and HMPP for 60 s were investigated as shown in Figure 2. Obviously, there is a remarkable improvement of the tensile strength of the UV-cured materials initiated by HBSMI in comparison to that of the coatings prepared with the same PUA initiated by HMPP. Especially for the UV-cured materials prepared from PUA with 20 wt% TMP, the tensile strength and the strain at break increased from 6.81 MPa to 12.14 MPa and from 43.0% to 71.9% respectively. The fraction of improvement for the tensile strength and the strain at break is as high as 78.9% and 67.2%, respectively. The improvement of the mechanical performance of UV-cured material may be attributed to the better solubility and compatibility of HBSMI than that of HMPP [25].

### 3.3. The Effect of Different Amount of HBSMI on the UV-Cured Coatings

The amount of HBSMI is one of the significant factors for UV-cured coatings as shown in Table 4. When m(HBSMI)/m(*PUA*) is only 1 wt% and UV-curing time is 60 s, the degree of curing content of the coating can reach 97.7%. Generally, the degree of curing content of these coatings is as high as 92.5–98.6%. It denotes that the UV-initiate efficiency of the HBSMI is quite high. A lower degree of curing content of coating for a higher m(HBSMI)/m(*PUA*) may be attributed to the migration of residual or pyrolysis of HBSMI. The pencil hardness of the coatings increased from 6 H to 9 H if m(HBSMI)/m(*PUA*) increased from 1 wt% to 4 wt%. If m(HBSMI)/m(*PUA*) is higher than 5 wt%, a higher m(HBSMI)/m(*PUA)* will result in a lower pencil hardness of UV-cured coatings.

**Figure 3:**
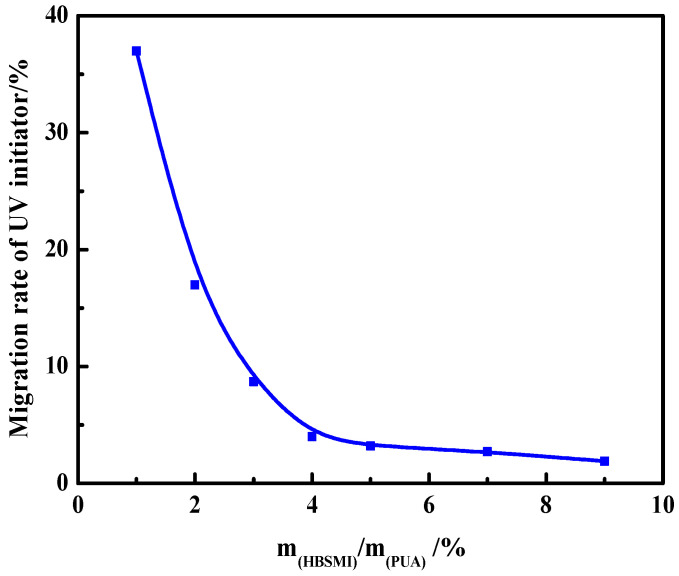
The migration rate of HBSMI from coatings cured by various amount of HBSMI.

High migration of UV initiator from UV-cured materials greatly limits the applications of UV-cured materials [10,13,14,15,16,17,18], especially in food packaging [14,19]. The migration rate of HBSMI obtained is evaluated and shown in Figure 3. Overall, the migration of HBSMI from the UV-cured materials gradually decreased from 37.0 wt% to 1.7 wt% with the increasing amount of HBSMI from 1 wt% to 9 wt% of PUA–3. If the amount of HBSMI is in the range of 4–9 wt% of PUA–3, the decreasing of migration is no longer obvious.

### 3.4. The Effect of Different HBSMI on the UV-Cured Coatings

The three-dimension chemical structure of HBSMI can be adjusted by variation in the molar ratio of the triethoxysilane with HMPP substitute obtained and NPG. Table 5 exhibits the influence of different HBSMI on the UV-cured coatings cured by UV for 60 s. Though the degree of curing content of these coatings is very close (97.1–98.6%), the degree of curing content for the coating prepared with HBSMI–5 is the highest while the migration rate of HBSMI–5 is the lowest. So HBSMI–5 is chosen to investigate the UV-curing behavior of the coating.

### 3.5. The Performance of UV-Cured Coatings Initiated by HBSMI

#### 3.5.1. The Transmittance of UV-Cured Coatings

**Figure 4:**
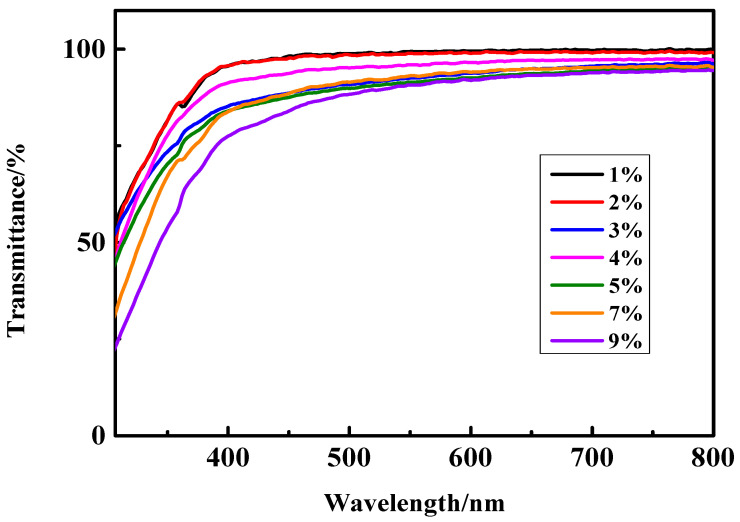
The transmittance of the cured coatings prepared with different amount of HBSMI–5.

Materials with high transmittance can be applied in optical devices [1,2,4,5,33] and there are still some challenges in development of coatings with high transmittance [1,2,4,5]. The transmittance of the coatings prepared with different amount of HBSMI–5 was studied as shown by Figure 4. The transmittance of the coatings is in the range of 80–99.5% (400–800 nm), which decreases with the increment of the amount of HBSMI–5. Generally, the lower the amount of HBSMI–5 is, the higher the transmittance of the coatings will be, which may be due to the better solubility and compatibility of a relative lower amount of HBSMI–5.

#### 3.5.2. Thermal Stability of UV-Cured Coatings

Thermal stability is an important impact factor for the materials used under elevated temperature. Thermal stability analyzed by TGA analysis for the coatings prepared with various HBSMIs was shown in Figure S5. For these coatings investigated, the initial decomposition temperature (T_d_5%) ranges from 288.7 °C to 292 °C. Overall, the variation of the HBSMIs has little effect on the thermal stability of UV-cured coatings.

#### 3.5.3. The UV Resistance of UV-Cured Coatings

**Figure 5:**
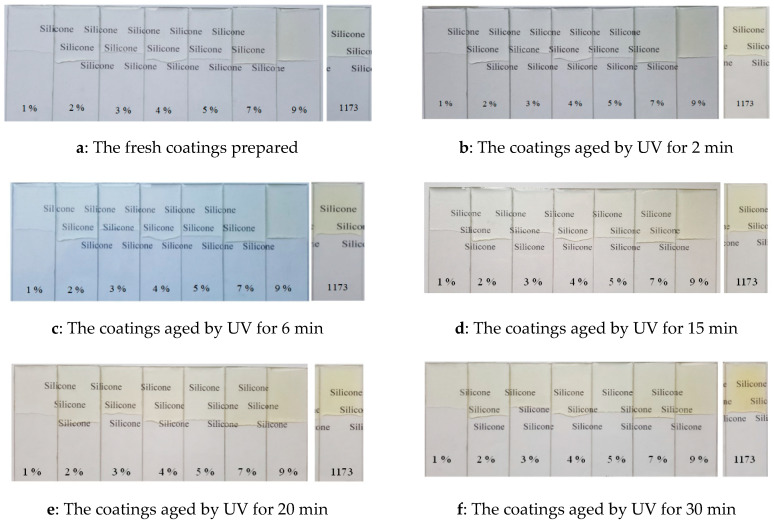
The photos of the coatings prepared with various amount of HBSMI–5 and 4 wt% of HMPP before and after being aged by UV.

When the coatings are exposed to UV radiation, the residual UV initiator will be photolyzed or the cured polymer will be cross-linked, which will lead to a poor performance of materials to some extent. So, the UV resistance of coatings is crucial for their applicability, especially under potentially harsh environmental conditions [20,24,39]. The obvious negative manifestation of transparent coatings exposed to UV radiation is turning yellow, therefore, the coatings prepared with various amount of HBSMI and 4 wt% of HMPP were aged by UV in an UV test chamber of 10.6 mw.cm⁻^2^ and the photos of these coatings were taken to record the process of turning yellow (Figure 5). It can be seen from Figure 5a that the fresh coatings prepared with various amount of HBSMI are transparent and colorless, while the fresh coating prepared with 4 wt% of HMPP is slightly yellow. The coatings prepared with various amount of HBSMI also turn slightly yellow when they are aged for about 15 min (Figure 5d). On the contrary, the coating prepared with 4 wt% of HMPP will turn yellow only when aged for 2 min. For the coatings prepared with various amount of HBSMI–*5*, a further increment of UV aging time to 20–30 min will obviously lead to turning yellow (Figure 5e,f). In brief, the coatings prepared with HBSMI have relatively better UV resistance ability.

## 4. Conclusions

In this paper, a kind of hyperbranched silicone containing macrophotoinitiators HBSMIs based on HMPP were synthesized and the UV-curing behaviors were investigated in UV-cured transparent polyurethane-acrylate coatings. The transmittance of the coatings is in the range of 80–99.5% (400–800 nm). Compared with HMPP, HBSMIs show fairly high UV-initiating efficiency because when the amount of HBSMI and HMPP is the same as 4 wt% of PUA, the degree of curing content and pencil hardness of the coatings cured by HBSMI for the same time are much higher than those coatings cured by HMPP respectively. The migration of HBSMI from the UV-cured materials is fairly low, which can be as low as 1.7–6.0 wt%. Obviously, there is a remarkable improvement of the tensile strength of the UV-cured materials initiated by HBSMI in comparison to that of the coatings prepared with the same PUA initiated by HMPP. Especially for the UV-cured materials prepared from PUA with 20 wt% TMP, the tensile strength and the strain at break increased from 6.81 MPa to 12.14 MPa and from 43.0% to 71.9% respectively. The fraction of improvement for the tensile strength and the strain at break is as high as 78.9% and 67.2%, respectively. The coatings prepared with HBSMI have better UV resistance ability because they turn slightly yellow when they are aged for about 15 min while the coating prepared with 4 wt% of HMPP will turn yellow only when aged for 2 min. The silicone containing hyperbranched macrophotoinitiators based on HMPP will be one of good initiators to improve the UV-initiating efficiency of the UV-curing system, reduce the migration of UV initiator from cured material, and improve the mechanical and UV resistance performance of the UV-cured materials.

## Figures and Tables

**Table 1:** The amount of raw materials for preparation various of polyurethane-acrylate (PUAs).

Entry	Sample Name of PUAs	The Molar Ratio of TMP to PTMG	PTMG/g	TMP/g	IPDI/g	HPA/g
1	PUA–1	10:100	200	1.342	63.797	49.738
2	PUA–2	15:100	200	2.013	68.910	51.901
3	PUA–3	20:100	200	2.683	72.244	56.226
4	PUA–4	25:100	200	3.354	76.245	59.110

Conditions: The amount of DBTDL is 0.2 wt% of PTMG.

**Table 2:** The amount of raw materials for preparation various of 2-hydroxy-2-methyl-1-phenyl propanone (HMPP).

Entry	Sample Name of HBSMIs	Molar Ratio of Triethoxysilane with HMPP Substitute	Triethoxysilane with HMPP Substitute/g	NPG/g
1	HBSMI–1	1:1.5	40.736	15.615
2	HBSMI–2	1:1.6		16.656
3	HBSMI–3	1:1.7		17.697
4	HBSMI–4	1:1.8		18.738
5	HBSMI–5	1:2.0		20.820
6	HBSMI–6	1:2.2		22.902

**Table 3:** Effect of UV-cured time on the properties of coatings.

Entry	Curing Time/s	Pencil Hardness of Coatings Cured with Different UV Initiators	
		HBSMI	HMPP
1	10	4H	6B
2	20	5H	6B
3	30	6H	6B
4	40	7H	2H
5	50	8H	3H
6	60	9H	9H
7	70	9H	9H

Conditions: PUA is PUA–3 and the amount of UV initiator is 4 wt% of PUA–3.

**Table 4:** Effect of amount of HBSMI on the UV-cured coatings.

Entry	mHBSMI/m*PUA*/%	Degree of Curing Content/%	Pencil Hardness
1	1	97.7	6H
2	2	97.5	7H
3	3	98.0	8H
4	4	98.6	9H
5	5	96.1	9H
6	7	94.3	8H
7	9	92.5	8H

Conditions: PUA is PUA–3. HBSMI is HBSMI–5 and the coatings were cured for 60 s.

**Table 5:** Effect of different macrophotoinitiator on the UV-cured coatings.

Entry	Sample of HBSMI	Degree of Curing Content/%	Migration Rate of HBSMI/%	Pencil Hardness
1	HBSMI–1	97.7	7.5	5H
2	HBSMI–2	97.7	3.3	5H
3	HBSMI–3	97.1	5.5	6H
4	HBSMI–4	97.2	7.3	8H
5	HBSMI–5	98.6	4.0	9H
6	HBSMI–6	98.6	5.5	7H

Conditions: PUA is PUA–3. HBSMI is 4 wt% of PUA–3 and the coatings were cured for 60 s.

## Supplementary Materials

The following are available online at https://www.mdpi.com/2073-4360/12/12/3005/s1. Figure S1: ^1^H–NMR for HBSMI. Figure S2: ^29^Si–NMR for HBSMI. Figure S3: SEC analysis for HBSMI. Figure S4: FT–IR spectra of coatings cured for various time initiated by HBSMI. Figure S5 and Table S1: TGA analysis for the cured coatings prepared with various of HBSMIs and 4 wt% of HMPP.
