# Supplementary material for: High Efficiency and Low Migration Hyperbranched Silicone Contain Macrophotoinitiators for UV-Cured Transparent Coatings
Source: Polymers (Basel). 2020 Dec 16;12(12):3005. doi: 10.3390/polym12123005 (PMC7766499; doi:10.3390/polym12123005)
Supplement: Supplementary file 1 [file polymers-12-03005-s001.pdf]

Supporting Information for

High Efficiency and Low Migration Hyperbranched Silicone Contain Macrophotoinitiators for UV Cured Transparent Coatings

*Yunxin Fan, Yan Song, Na He, Fei Cheng, Xiaojiao Jiao, Guoqiao Lai, Xilin Hua and Xiongfa Yang**

(Key Laboratory of Organosilicon Chemistry and Material Technology of Education Ministry, College of Material, Chemistry, and Chemical Engineering, Hangzhou Normal University, Hangzhou 311121, China.)

* Author for the correspondences: Xiongfa Yang, Email:yangxiongfa@hznu.edu.cn

Number of Pages:7

Number of Figures: 5

Number of Tables: 1

1. ^{1}H–NMR for HBSMI

^{1}H–NMR spectrum (400 Hz, shown in Figure S1):3.96–3.81, 3.80–3.60 and 3.56–3.40 ppm are assigned to the protons of $–SiOC\boldsymbol{H}_2C(CH_3)_2C\boldsymbol{H}_2OSi–$ & $HOCH_2C(CH_3)_2C\boldsymbol{H}_2OSi–$, $HOC\boldsymbol{H}_2C(CH_3)_2CH_2O–$, $–SiOC\boldsymbol{H}_2CH_3$, respectively. 7.52–7.15 ppm is assigned to the proton of $–C_6\boldsymbol{H}_5$ in HMPP substitute. 1.0–0.70 ppm is assigned to the protons of $–C\boldsymbol{H}_3$ in HMPP substitute, $–SiOCH_2C\boldsymbol{H}_3$, $–SiOCH_2C(C\boldsymbol{H}_3)_2CH_2OSi–$ & $HOCH_2C(C\boldsymbol{H}_3)_2CH_2OSi–$. 1.57–1.40, 2.43–2.30 and 1.19–1.0 ppm are assigned to the protons of $–SiCH_2C\boldsymbol{H}_2CH_2NCO–$, $–SiCH_2CH_2C\boldsymbol{H}_2NCO–$ and $–SiC\boldsymbol{H}_2CH_2CH_2NCO–$, respectively.

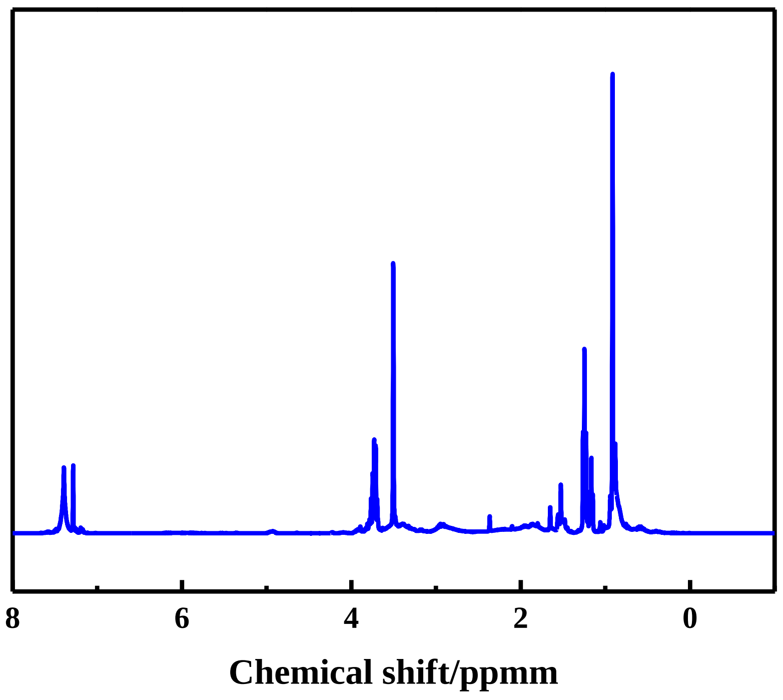

Figure S1 ^{1}H–NMR for HBSMI

2. ^{29}Si–NMR for HBSMI

^{29}Si–NMR spectroscopy of the HBSMI obtained as shown in Figure S2.The chemical shifts at –59.3 and –68.1 ppm can be attributed to the complete branched and the incomplete branched Si respectively, which is according to reference S1 and S2. The broad peak in the range of -75.0 ppm – -125.0 ppm is assigned to the Si in quartz NMR tube.

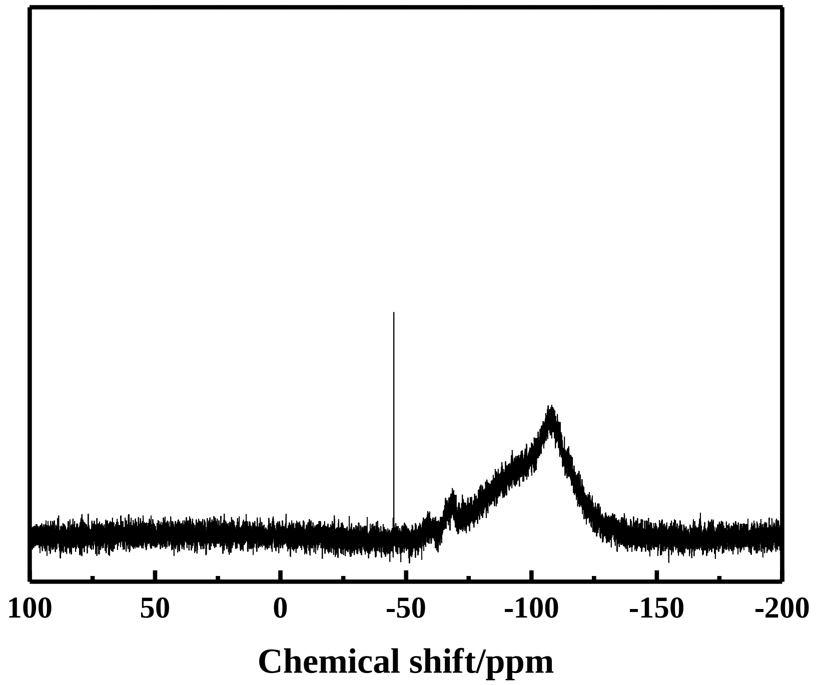

Figure S2 ^{29}Si–NMR for HBSMI

3. SEC analysis for HBSMI

Figure S3 reveals the results of SEC analysis for HBSMI prepared. It can be seen that the average number average molecular weight (Mn) of HBSMI is about 1.54×10^{6}, molecular weight distribution is PDI=2.90 and the α constant of Mark–Houwink–Sakurada is 0.178(±3.64%), which means the HBSMI obtained is a kind of hyperbranched polymer.

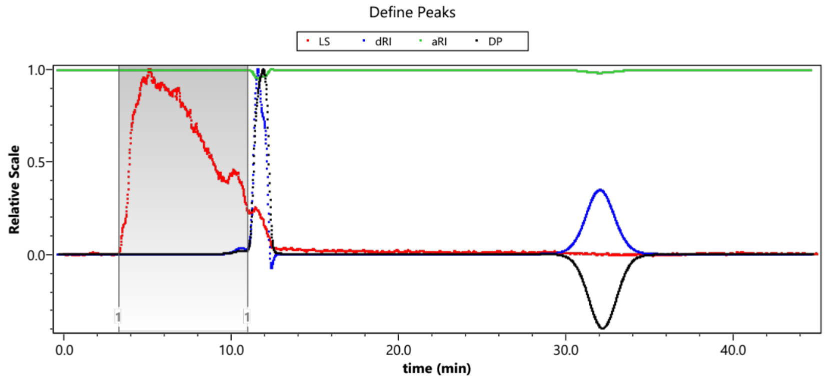

Configuration

Notes:

Concentration Source: RI
Flow Rate: 1.000 mL/min

Light Scattering Instrument: HELEOS
- **Cell Type:** Fused Silica
- **Wavelength:** 661.0 nm
- **Calibration Constant:** 3.1976×10^{-5} 1/(V cm)

RI Instrument: rEX

Viscometer: ViscoStar
- **Dilution Factor:** 0.5

Solvent: THF
- **Temperature Correction Enabled:** yes
- **Refractive Index:** 1.402

Processing

Collection Time: Tuesday, July 14, 2020 16:01:52 PM
Processing Time: Thursday, October 22, 2020 15:59:12 PM

Peak settings:

**Peak Name**	Peak 1
**Peak Limits (min)**	3.313 - 10.985
**Light Scattering Model**	Zimm
**Fit Degree**	1
**dn/dc (mL/g)**	0.1850
**A2 (mol mL/g²)**	0.000
**UV Ext. Coef. (mL/(mg cm))**	0.000
**Viscometry Model**	Huggins
**Huggins Equation Parameter**	0

Kraemers Equation Parameter 0

Molar Mass & Radius from LS:

Enabled Detectors: 3 4 5 6 7 8 9 10 11 12 13 14 15 16 17 18

Results Fitting Procedure:

Data	Fit Model	Degree	R^2	Extrapolation
Molar Mass	None	n/a	n/a	none
Rms Radius	None	n/a	n/a	none
Mean Square Radius	None	n/a	n/a	none
Hydrodynamic Radius (Q)	None	n/a	n/a	none

Results

Peak Results

	Peak 1
**Hydrodynamic radius (v) moments (nm)**	
rh(v)n	1.820 (±1.583%)
rh(v)w	2.149 (±3.033%)
rh(v)z	13.266 (±29.494%)
**Masses**	
Calculated Mass (μg)	108.38
Mass Recovery (%)	n/a
Mass Fraction (%)	100.0
**Molar mass moments (g/mol)**	
Mn	6.878×10^3 (±3.965%)
Mp	5.449×10^3 (±3.832%)
Mv	8.565×10^3 (±0.722%)
Mw	1.992×10^4 (±2.413%)
Mz	1.226×10^6 (±3.647%)
**Polydispersity**	
Mw/Mn	2.896 (±4.641%)
Mz/Mn	178.264 (±5.387%)
**rms radius moments (nm)**	
rn	83.2 (±2.4%)
rw	84.6 (±2.2%)
rz	108.7 (±1.0%)
**Intrinsic viscosity moments (mL/g)**	
[η]n	5.884 (±2.376%)
[η]w	6.29 (±5.21%)
[η]z	22.819 (±77.254%)

Mark-Houwink-Sakurada a: 0.178 (±3.635%)
Mark-Houwink-Sakurada K: 1.238 (±5.722%) mL/g

Mn=6.88×10^3 Da, PDI=1.90, α=0.178, k=1.238

Figure S3 SEC analysis for HBSMI

4. *FT*–IR spectra of coatings cured for various time initiated by HBSMI

FT–IR spectra of coatings cured for various time initiated by HBSMI shown in Figure S4. Even the coatings were cured only for 10 s, the characteristic absorption peaks of acrylate in PUA is vanished.

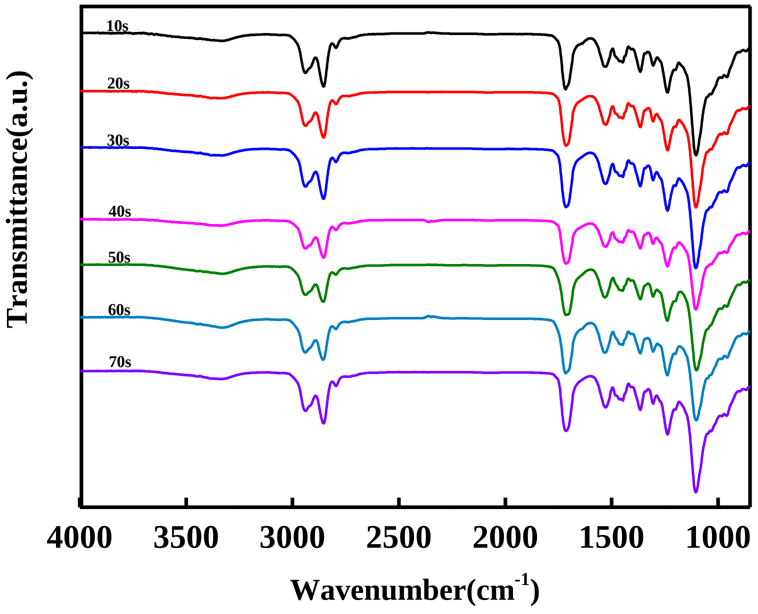

Figure S4 *FT*–IR spectra of coatings cured for various time initiated by HBSMI

5. TGA analysis for the cured coatings prepared with various of HBSMIs and 4 wt% of HMPP

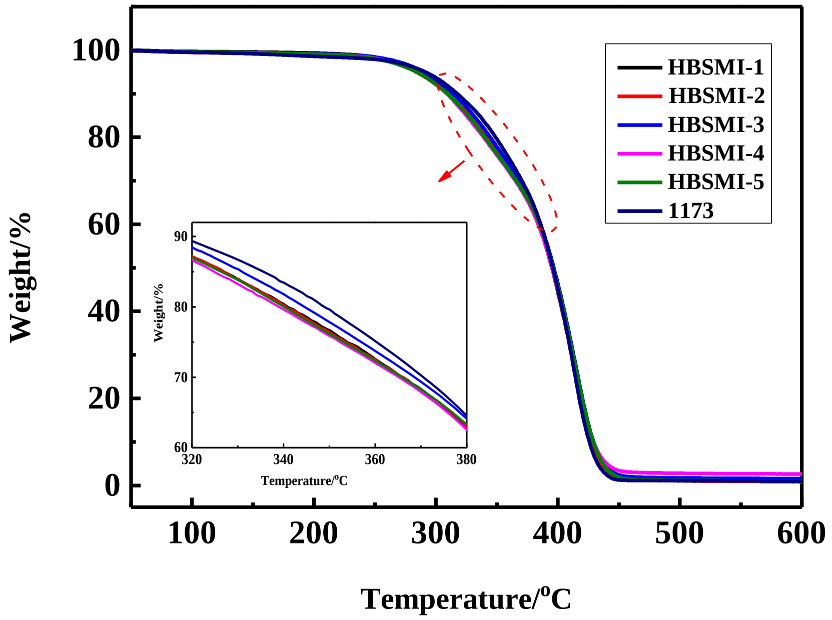

Figure S5. The TGA analysis for the cured coatings prepared with various of HBSMIs and 4 wt% of HMPP.

Table S1 The TGA analysis for the cured coatings prepared with various of HBSMIs and 4 wt% of HMPP.

Entry	HBSMIs	Td 2%/°C	Td 5%/°C	Td 15%/°C
1	HBSMI–1	258.0	289.0	327.1
2	HBSMI–2	258.0	289.0	327.5
3	HBSMI–3	253.5	291.0	331.0
4	HBSMI–4	251.8	289.5	324.5
5	HBSMI–5	250.7	284.0	327.3
6[a]	–	234.5	291.4	335.5

a: Prepared with 4 wt% of HMPP

REFERENCES

S1. Niu, S.; Yan, H.X.; Chen, Z.Y.; Li, S.; Xu, P.L.; Zhi, X.L. Unanticipated Bright Blue Fluorescence Produced from Novel Hyperbranched Polysiloxanes Carrying Unconjugated Carbon–Carbon Double Bonds and Hydroxyl Groups. Polym. Chem. 2016, 7,3747.

S2. Niu, S.; Yan, H.X.; Li, S.; Tang, C.; Chen, Z.Y.; Zhi, X.L.; Xu, P.L. A Multifunctional Silicon–Containing Hyperbranched Epoxy: Controlled Synthesis, Toughening Bismaleimide and Fluorescent Properties. J. Mater. Chem. C 2016, 4(28):6881–6893.
